# University of Louisville

**Published:** 1854-03

**Authors:** 


### UNIVERSITY OF LOUISVILLE.

THE eighth session of the Medical Department of the University of Louisville, and the seventeenth of the School, closed with a public Commencement, in the Chemical lecture room, on the evening of the 3d inst., in presence of the officers of the Institution and of a large assemblage of ladies and gentlemen. The exercises were opened with prayer by the Rev. Dr. W. L. BRECKINRIDGE, after which the President, *pro. tem.*, of the Board of Trustees, JAMES SPEED, ESQ., proceeded to confer the degree of *Doctor of Medicine*, upon seventy-nine young gentlemen, who had passed satisfactory examinations before the Medical Faculty, and complied with the other requirements of the University. The honorary degree of *M. D.* was conferred upon Dr. J. P. EPPERSON, of Pulaski, Tenn., and the *ad eundem* degree upon Dr. PLEASANT B. ROBINSON, of Huntsville, Ala., a graduate of the Cincinnati Medical College. Prof. SILLIMAN pronounced the Valedictory Address to the Graduates on behalf of the Faculty. It was an address of uncommon merit, abounding in just thoughts, and elevated sentiments, forcibly and eloquently expressed. The subject was Education and the duties of educated men. Prof. S. urged the importance of a higher standard of education in our country, as a means of correcting the tendency of the public mind to embrace quackery in all its forms. He showed that the effect of thorough scientific knowledge was to suppress superstition. In order to the creation of a class of scholars profoundly skilled in all the departments of human knowledge, he insisted that it was neeessary that we should have an institution of a higher order than any now existing in our country—a *National University*, where the graduates of our Colleges and Universities might repair to perfect themselves in their chosen departments of science or art.

At the close of his address, Prof. SILLIMAN announced that he was then performing his last official act in the University of Louisville, having already tendered his resignation to the Board of Trustees. He would not attempt, he said, to describe the contest of feelings through which he had passed in reaching that determination. He had experienced great difficulty in coming to it, but Providence seemed to have indicated that it was his duty to return to the theatre of his earlier labors. He spoke of the perfect harmony that had prevailed in the Faculty, aud of his pleasant relations with all the members who had belonged to it during his connection with the school, in handsome and fitting terms. He returns to New Haven, to occupy the place of his distinguished father, in Yale College, and we have no doubt that he will satisfy the highest expectations of his friends in his new sphere of action. To the ample stock of chemical knowledge, and his expertness of manipulation in the laboratory with which he came to the University, he now adds several years of the most valuable experience as a public teacher. He bears with him the confidence and best wishes of his colleagues and pupils.

The session which terminated with these exercises was a pleasant and entirely prosperous one. The class was larger than any preceding one since the year 1849, numbering two hundred and eighty-seven. The graduates presented a fine appearance, and among them are men capable of reaching any point they desire in their profession. No class, we are persuaded, ever retired from the institution more favorably impressed with it as a seat of medical learning. The lectures, the hospital, the University Clinic, the dissecting rooms, all gave satisfaction. It is gratifying that the school, for a long time past the great school of the Mississippi Valley, has maintained its relative rank among the medical schools of the United States notwithstanding the temporary declension of its classes, being still the third in point of numbers; and the causes which affected it injuriously having ceased to operate, its friends have a right to indulge in expectations of its continued prosperity. The Board of Trustees have a fund at their command which enables them to make large annual additions to the library, museum, and all the apparatus of medical instruction. The reputation of the School is such, that they have no difficulty in filling vacancies, when they occur, with men of repute. The Chair of Chemistry now vacant, they are resolved to fill with a Chemist of distinction.

Prof. FLINT has sailed for Europe where he will spend the vacation, visiting the hospitals of the chief cities, and making purchases for the University. He expects to return early in October.

